# The Invasive Bank Vole (*Myodes glareolus*): A Model System for Studying Parasites and Ecoimmunology during a Biological Invasion

**DOI:** 10.3390/ani11092529

**Published:** 2021-08-28

**Authors:** Andrew McManus, Celia V. Holland, Heikki Henttonen, Peter Stuart

**Affiliations:** 1 Department of Biological and Pharmaceutical Sciences, Munster Technological University, Clash, V92 CX88 Tralee, Ireland; peter.stuart@mtu.ie; 2 Department of Zoology, Trinity College Dublin, the University of Dublin, College Green, D02 PN40 Dublin, Ireland; cholland@tcd.ie; 3 Wildlife Ecology, Natural Resources Institute Finland (Luke), FI 00790 Helsinki, Finland; ext.heikki.henttonen@luke.fi

**Keywords:** biological invasion, emerging infectious diseases, parasite dynamics

## Abstract

**Simple Summary:**

The recent emergence of SARS-CoV-2 has highlighted the recent increase in Emerging Infectious Diseases since the 1940s. This has made evident the need for wildlife studies investigating pathogen dynamics in wildlife species. Rodents have proved excellent models, in both laboratory and natural settings for studying disease dynamics. Due to the single introduction point, continuous spread and presence of baseline data, we propose that the recent invasion of *Myodes glareolus* in Ireland can be used as a model system to understand the changes in helminth species during a biological invasion. Through long-term studies using this invasive species as a model, we will be able to fill large knowledge gaps surrounding the area of pathogen dynamics in wild populations.

**Abstract:**

The primary driver of the observed increase in emerging infectious diseases (EIDs) has been identified as human interaction with wildlife and this increase has emphasized knowledge gaps in wildlife pathogens dynamics. Wild rodent models have proven excellent for studying changes in parasite communities and have been a particular focus of eco-immunological research. Helminth species have been shown to be one of the factors regulating rodent abundance and indirectly affect disease burden through trade-offs between immune pathways. The *Myodes glareolus* invasion in Ireland is a unique model system to explore the invasion dynamics of helminth species. Studies of the invasive population of *M. glareolus* in Ireland have revealed a verifiable introduction point and its steady spread. Helminths studies of this invasion have identified enemy release, spillover, spillback and dilution taking place. Longitudinal studies have the potential to demonstrate the interplay between helminth parasite dynamics and both immune adaptation and coinfecting microparasites as *M. glareolus* become established across Ireland. Using the *M. glareolus* invasion as a model system and other similar wildlife systems, we can begin to fill the large gap in our knowledge surrounding the area of wildlife pathogen dynamics.

## 1. Emerging Infectious Diseases and the Need for Wildlife Models

**Figure 1:**
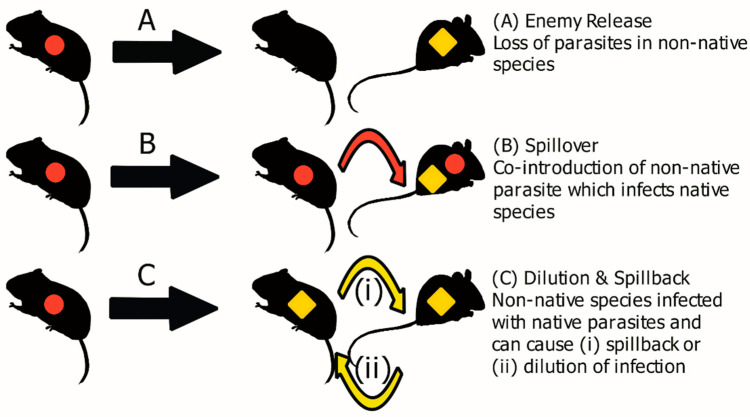
Disease dynamics of a biological invasion. Parasites introduced with the non-native species are indicated by red circles, while parasites from the native host are indicated by yellow diamonds. Adapted from Loxton [26] and Hatcher and Dunn [28].

In 2020, a global public health emergency was declared by the World Health Organisation (WHO) following the detection of a new zoonotic disease, similar to the SARS-CoV virus [1]. This disease, called COVID-19, has caused an ongoing global pandemic resulting in over 3.8 million deaths worldwide [2]. The COVID-19 disease sparked an unprecedented scientific effort including the rapid identification and genome sequencing of the virus, and the swift development of efficacious vaccines [3,4,5]. Despite this, relatively little is known about the wildlife origins of the SARS-CoV-2 virus. Although evidence supports an origin from a bat species, it is not known exactly from what species of bat is associated or if there was an intermediate host involved between bats and humans [6]. This prevents the investigation of any sylvatic epidemiological or host factors that may have led to the spillover to humans. This pandemic is not an isolated event, the incidence of emerging infectious diseases (EIDs) has increased since the 1940s [7]. A review by Jones et al. [7] demonstrated that 60% of EIDs originate from wildlife. These EID events further spotlight the need for investigation into wildlife pathogens. EID events not only pose a risk to humans, but also can be detrimental to indigenous wildlife and livestock [8,9,10]. The increased frequency of EIDs has been primarily attributed to the increasing rate of environmental change, caused by processes such as human development, habitat destruction, lowering habitat diversity and biological invasions [11,12,13]. Invasive species can also harbour zoonotic parasites [14], such as the golden apple snail (*Pomacea canaliculata*), a freshwater mollusc which harbours *Echinostoma revolutum, Angiostrongylus cantonensis* and *Gonathstoma spinigerum*, helminths known to impair human health [15]. Similarly, it has been suggested that the bioinvasion of the grey squirrel (*Sciurus carolinensis*), has caused the spillover of Squirrelpox (*Parapoxvirus*) to indigenous wildlife, such as the native red squirrel (*Sciurus vulgaris*) [16,17,18]. The introduction of pathogens can occur through co-invasion with non-native host species, these pathogens can then infect native species, a process known as pathogen spillover (Figure 1B) [19]. The emergence of these new pathogens from invasive species, once established, can cause increased disease risk for both humans and animals [20]. The introduction of exotic pathogens to immunologically naïve indigenous wildlife has been largely ignored by conservation biologists, possibly due to their cryptic nature [8]. Another process, known as spillback, can occur when an invasive species is a more competent host for native parasite species than a native host, resulting in an amplification of infection in native hosts (Figure 1C) [21,22]. Aside from pathogen spillover and spillback, during a biological invasion, non-native species can benefit from a reduction in parasitism, known as enemy release (Figure 1A), this can happen through various mechanisms, such as absence from the founder population, or unsuitable environmental conditions [14,23,24,25,26]. For example, the invasive rodent, *Mus musculus domesticus,* in Senegal has been shown to exhibit enemy release by demonstrating a low prevalence and abundance of gastrointestinal helminths in general and specifically, enemy release along its invasion route, with the helminth, *Aspiculuris tetraptera,* absent from populations at the invasion front [27]. This reduction in parasites has been postulated to enhance invasiveness by allowing a reallocation of resources from immune functions to dispersal and reproduction, known as the evolution of increased competitive ability theory (EICA) [23,24].

The example of cross-species transmission, increase in frequency of EIDs and ongoing human-wildlife interactions outlined above demonstrate a pressing need to increase our understanding of the role wildlife, ecology and inter-species interactions play in pathogen transmission and spillover [12,29,30]. Host diversity has been shown to have a positive effect on reducing the presence of pathogens in the environment, providing a dilution effect [22,31]. This dilution effect occurs through the presence of hosts with a lower competency for a specific pathogen, reducing the overall prevalence of that pathogen in competent hosts (Figure 1C) [32,33]. As the dilution effect has been frequently observed in studied bio-invasion they present an ideal system to study the dilution effect, in particular through longitudinal studies [22]. For example, a study by Tierney et al. [34] found that the invasive populations of the freshwater cyprinid dace (*Leuciscus leuciscus*) in Ireland caused a dilution effect in the native brown trout (*Salmo trutta*), reducing the abundance of the native helminth *Pomphorhynchus tereticollis* in *S. trutta* at sites where *L. leuciscus* was established longest. Consequently, studying wildlife and biological invasions can provide a unique insight into improving our understanding of pathogen dynamics and in-turn our comprehension of EIDs [12,29,31,35,36].

## 2. Cycling Rodent Populations as Wildlife Disease Models

In the past, experimental approaches have used laboratory rodents to study zoonotic-borne pathogens and describe stages of infection and transmission [37,38]. In comparison to the wild, laboratory settings can be highly regulated, with constant food and water supplies, reduced genetic diversity, controlled infection and known immune markers [38]. While these laboratory-based studies have their strengths for determining cellular-level responses and the structure of molecular pathways [39], relative to natural systems they fall short in identifying the influence of natural genetic variation and habitat diversity on disease spread and vaccination success [38,40]. For example, Voutilainen et al. [41] found that wild populations of the bank vole *Myodes glareolus* infected with Puumala Orthohantavirus manifested life-long periods of virus shedding compared to laboratory studies. Other disadvantages of laboratory studies include the lower genetic diversity of laboratory-reared inbred mice compared to wild populations [40]. Pedersen and Babayan [38], identified that the majority of knowledge on immune responses come from laboratory studies, showing an increasing need for ecological studies to expand our knowledge of immunology in wild populations, known as wild immunology or ecoimmunology. Rodents make up the largest group of mammals, consisting of ~1500 species, with large populations and wide distributions, short generation times and considerable laboratory-based knowledge [38,39]. This makes studying rodent pathogens and their natural ecology, an ideal model system for studying pathogens in wildlife populations and parasite communities in a natural setting, potentially identifying possible reservoirs of zoonotic diseases and pathogen dynamics during biological invasions [38,42].

As a group, rodent population dynamics have been thoroughly studied, with some populations having been shown to display population multi-annual fluctuations, known as population cycles, while others show less-extreme fluctuations seasonally and are considered non-cyclic [43,44,45]. These population dynamics have been shown to be a response to extrinsic factors, such as local environmental conditions, number of generalist or specialist predators and food availability [43,44,45,46,47,48,49,50]. It has been shown that parasites can affect fecundity and mortality rates of rodents [44,51,52]. In a laboratory setting, Scott [53] infected outbred CD1 mice with the nematode, *Heligmosomoides polygyrus*, and observed a reduction in host abundance. This was due to a high initial host mortality following infection, which Scott postulates could be what happens when a new parasite is introduced to a naïve host [53]. Following this, anthelmintic treatment was provided to the mice and the populations then recovered to original densities [53]. Scott [53] also acknowledged the need for their study to be extrapolated into wild rodent populations, to test if these findings hold true in natural systems. Likewise, wild populations of white-footed mice (*Peromyscus leucopus*) and deer mice (*Peromyscus maniculatus*) were treated with food supplementation and an anthelmintic drug, ivermectin, showed significantly reduced population crashes compared to control groups not receiving the treatment [54].

Studying cycling populations of rodents has also extended our knowledge of parasite dynamics. The changing population of rodents during each phase makes rodent cycles an informative wildlife model to study parasite epidemiology, with the sequential density fluctuations and corresponding resource availability and trophic interactions, allowing for the rigorous testing of hypotheses. For example Haukisalmi, Henttonen and Tenora [55] recorded that the peak *Heligmosomum mixtum* infection in bank voles and red voles (*Myodes rutilus*), occurred in winter, followed by a subsequent decline in spring, coinciding with vole maturation, followed by a new increase in old overwintered voles in summer. It was also found that *Heligmosomum sp*. and *Catenotaenia sp.* showed interspecific synchrony, despite their different life cycles, direct in *Heligmosomum* or via intermediate hosts [55]. Furthermore, Haukisalmi and Henttonen [56,57] found *H. mixtum* and *Heligmosomum glareoli* had a positive co-occurrence pattern, despite occupying the same microhabitat. These authors also noted that a negative interaction would be expected, however, the different feeding nodes and radial distribution of these two helminth species may account for their positive co-occurance [57]. Likewise, *Mastophorus muris* was shown to have a positive association with *Capillaria sp.*, however these species occupy different microhabitats, with *M. muris* present in the stomach and *Capillaria* in the small intestine [56]. Stien et al. [58] linked arctic fox (*Vulpes lagopus*) parasite abundance to the patchy local presence of the intermediate host, sibling vole (*Microtus levis*), showing that increasing distance from vole sites resulted in reduction in the prevalence of vole-transmitted cestodes, *Echinococcus multilocularis*, *Taenia crassiceps* and *Taenia polyacantha*, in arctic foxes. In years of lower sibling vole abundance, it was shown that there was a lower presence in the fox diet [58]. Similarly, increases in abundances of *Toxascaris leonina* and *Toxocara canis* in red fox (*Vulpes vulpes*) have proven to be proportionate to increases in rodent density [59]. 

An essential part of studying the epidemiology of EIDs in humans is through the understanding of disease cycles in wild host populations [60]. This topic has been extensively studied in rodents. Laakkonen et al. [61], showed that *Eimeria* infections cycle seasonally in three vole populations, *Microtus agrestis, Microtus oeconomus* and *M. glareolus*, with the highest peak being in autumn, corresponding to the high number of immunologically naïve juveniles, and the lowest in spring when most of the vole population had increased its immunity over winter. Similarly, Puumala orthohantavirus (PUUV) prevalence in *M. glareolus* populations cycles seasonally, however in contrast to the study outlined above by Laakkonen et al. [61], it was found that prevalence was highest in spring and lowest in late summer–early autumn [62]. The Spring peak is believed to be result of age-related accumulation of the virus over winter, while in Autumn, prevalence is diluted by the presence of juveniles at the end of the breeding season [62]. The ecology of PUUV in Europe is biome specific [63] in the boreal zone it is top down (specialist predation on hosts), while in the temperate zone, bank vole dynamics are driven by mast years (years of heavy seed crops), which were also accompanied by high human hantavirus incidence [64].

Rodents also prove to be excellent models for studying immunological variation in wild populations. A review by Ezenwa and Jolles, [65] highlights the impact of coinfection on disease dynamics, with helminths inducing T-helper cell type 2 (Th2) responses, which down-regulate T-helper cell type 1 (Th1) responses. Similarly, these authors note that some helminth species exploit regulatory T cells to suppress immune responses [65]. In addition, the presence of helminths has also been found to be a factor influencing Puumala hantavirus prevalence in rodents, with PUUV infection being positively associated with the presence of *H. mixtum* [66]. Guivier et al. [67] also found a negative correlation between proinflammatory responses TNF-α and MX2 genes with PUUV load. These authors also demonstrated that PUUV-infected voles demonstrated higher TNF-α expression compared to uninfected voles or voles co-infected with *H. mixtum* [67,68]. Similarly, during a study in Kielder Forest, Jackson et al. [69], showed two potential trade-offs in the field vole (*Microtus agrestis*), the first being a potential trade-off with immune expression between Th1 and Th2 immune responses in the field vole-Th2 transcription factor Gata-3 was significantly negatively associated with IFN-γ and IL-2 (Th1 mediators). The second potential trade-off was between breeding condition and the immune response, due to an increased allocation of resources to reproduction [69]. Trade-offs in immune responses have the potential to influence parasite communities and the infection risk and severity of viral diseases of human importance [66]. A recent study by Charbonnel et al. [70] found a bias in immune gene expression towards the upregulation of serpins, alipoproteins and proinflammatory cascades at sites recently invaded by the house mouse (*Mus musculus domesticus*), suggesting that phenotypic differentiation in immune response between conspecific hosts along invasion routes could be mediated by changes in parasite infections. The knowledge from these studies combined prove that rodents provide the potential to be used as model systems to understand pathogen dynamics, and the complex relationship between parasites and host immune responses in wild populations.

## 3. Factors Present in the Irish Invasion System Making It an Ideal Study System for Parasite Dynamics

Ireland has a relatively depauperate community of small mammals in contrast to mainland Europe, with some debate regarding what species are truly native as opposed to those species that have been introduced by humans and hence been naturalised [71]. However, it is widely accepted that the wood mouse (*Apodemus sylvaticus*) is native and *M. glareolus* is non-native [72,73]. Evidence supports the introduction of *M. glareolus* into Ireland around the 1920s during construction of the Shannon Hydroelectric Plant when equipment was imported from Germany [74]. Evidence for the vole introduction was demonstrated by the close relationship of mtDNA sequences of Irish and German populations of *M. glareolus* [74]. *M. glareolus* in Ireland is a particularly informative model because the vole population has an identified core population, expansion front and area beyond the expansion front where *M. glareolus* is absent [22,75]. The vole population has also been shown to have a constant rate of spread ranging from 2.23–2.63 km per year in unconstrained areas, creating an invasion gradient from core established sites to more recently invaded frontier sites [22,76,77]. Most invasive species are under current eradication plans, however, there has never been an attempt to eradicate *M. glareolus* from Ireland [22,76].

White et al. [75] studied the allele frequency of *M. glareolus* during its expansion in Ireland, and found that there was an overall loss of vole genetic diversity along the transects (relevant to the core) due the strong selection pressures present during an expansion. These authors also found that the eastern expansion had lost the least diversity, possibly because of a lack of barriers to dispersal, and that the northern and north-eastern groups had lost the most, possibly due to the numerous barriers to dispersal such as the River Shannon and presence of unsuitable bog habitat [75]. However, pooling all three transects together, the genetic diversity was nearly as high as that described for the founder population, showing that each transect had different selection pressures, causing selection for different genes [75]. White et al. [75] also identified the selection for genes of immunological function in *M. glareolus* in sites furthest from the identified point of introduction. Stuart et al. [22] suggested that the immunogenetic changes observed during the White et al. study [75] may be related to the variation in helminth infection witnessed in *M. glareolus* across the invasion gradient.

Early studies on the helminths of *M. glareolus* and *A. sylvaticus,* respectively, on Ross Island in Ireland, identified the species of helminth parasites present and included cestodes, nematodes and trematodes (Table 1) [78]. Similar studies in the north of Ireland, documented nine helminth species in the alimentary canal of *A. sylvaticus*, sampled during the period of November 1978 to October 1981, with additional parasite surveys conducted in 1980 and 1985 (Table 1), with *Capillaria murissylvatici*, *Heligosomoides polygyrus*, *Syphacia stroma* and *Corrigia vitta* showing cyclic seasonal patterns [79,80,81]. Likewise, due to these seasonal patterns, the authors demonstrated that samples collected during similar seasons between years showed more similarities than samples collected within the same year [81].

More recent studies on the Irish population of *M. glareolus* revealed a depauperate helminth community compared to their native range, with only three species of helminth recovered, a phenomenon known as enemy release (Figure 1A), possibly aiding the spread of voles across Ireland (Table 1) [22,82,83]. Most noticeable was the absence of *H. mixtum* in Irish populations, a helminth often found in European *M. glareolus* [82,84]. Loxton et al. [82] proposed that the enemy release resulted from the loss of helminth species from the indigenous range in the founder population of *M. glareolus* during translocation and establishment in Ireland, and the lack of native helminth species obtained following establishment. Furthermore, Loxton et al. [82], found that *M. glareolus* carried a relatively low number of helminth species in comparison to the sympatric native *A. sylvaticus*. Furthermore, *M. glareolus* at the expansion front were found to be less parasitised than *M. glareolus* in longer established areas, with the abundance of *Aspiculuris tianjinensis* being highest at the core [22]. An increased prevalence of *Aonchotheca murissylvatici* was observed at core and invaded sites, which is believed to be amplified by the increased competence of *M. glareolus* as a host [22,83]. Loxton et al. [83] also detected *Taenia martis* in *A. sylvaticus* for the first time, at sites invaded by *M. glareolus* suggesting the possibility of co-invasion. In addition, more recent studies revealed *T. polyacantha* infection in M. glareolus and *A. sylvaticus* at invaded sites [22]. However, *M. glareolus* is the intermediate host and it is suggested these helminths may have been overlooked previously in studies due to their presence outside the digestive tract, residing in the thoracic and abdominal cavities [22]. A comparable study by Perkins et al. [77] found *M. glareolus* from the invasion front to have lower parasite abundances compared to conspecifics at core sites.

There is evidence to suggest that, overall, the presence of *M. glareolus* in Ireland has a dilution effect on the helminth community in *A. sylvaticus*, with a lower Brillouin’s Index of Diversity at the core invaded sites [22]. *M. glareolus* has also resulted in the dilution of *Bartonella* in *A. sylvaticus* with a lower prevalence observed in sites with high *M. glareolus* density, as *M. glareolus* appears to be resistant to native strains [85]. Previously, studies had shown *M. glareolus* to have a strong dilution effect on *Syphacia stroma* in *A. sylvaticus* [83]. However, more recently, *S. stroma* was found to show higher abundances in *A. sylvaticus* at core sites, suggesting it could be taking advantage of the lower helminth species diversity [22]. These observations also indicate that helminth communities show a lag effect during a biological invasion, changing as the invasive host becomes more established [22]. Both studies found the abundance of *Skrjabinotaenia lobata* to be higher in *A. sylvaticus* at core sites [22,83]. Both species*, S. stroma* and *S. lobata*, are known to compete for resources and are postulated to be benefitting from the lower species diversity [22]. Seasonal patterns were also detected for *S. stroma*, showing the lowest prevalence in autumn [22]. Kronfeld-Schor et al. [86] highlights the need for more wildlife studies, focusing on the seasonality of infectious diseases, and host immune responses.

The addition of a second invasive mammal, the greater white-toothed shrew (*Crocidura russula*), has had a positive, synergistic effect on *M. glareolus* abundance, with a negative effect on *A. sylvaticus* numbers and the complete perturbation of the native pygmy shrew (*Sorex minutus*), a process known as invasional meltdown [87]. This more recent introduction, discovered in 2007, was estimated to have happened around 2001 [88], and *C. russula* has been shown to displace other shrew species in central Europe [89]. The factors outlined here, including dilution, spillover, spillback, enemy release and the recent discovery of invasional meltdown, demonstrate that the Irish *M. glareolus* population is an informative model system to study the dynamics of an invasive species, including disease, and their effects on native fauna through longitudinal and spatiotemporal studies.

## 4. Opportunities Presented by the Bank Vole Invasion Model

**Figure 2:**
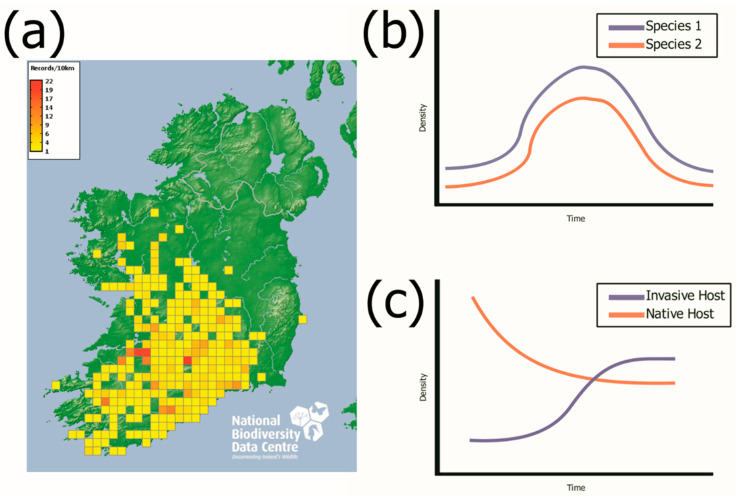
Current range of the bank vole (*M. glareolus*) and hypothetical population dynamics at core and expansion front sites. Part (**a**) shows the current distribution of the *M. glareolus* in Ireland. Part (**b**) provides an illustration of the multi-annual population cycle of two host species. Part (**c**) provides an illustration of the population dynamics of two host species when the population of the native host species declines as the population of the invasive host species increases. Part (**a**) taken with permission from the National Biodiversity Data Centre, Ireland [92].

Studies on Irish populations of *M. glareolus* have managed to pinpoint the possible origin of the invasion in Ireland, probable source population and the expansion rate throughout the island [74]. While our knowledge of helminth communities in *M. glareolus* has increased over the past century [84], we still have the opportunity to explore new avenues with the Irish population, as a model system for studying disease during a species invasion [22]. Previous studies have used spatiotemporal studies to examine parasite dynamics during a biological invasion [22,82,83]. However, longitudinal studies are required to fully understand the dynamic nature of parasite infections [22,60,90]. As seen in Figure 2a, *M. glareolus* currently occupies about 40% of the island of Ireland, mainly found in the Munster and Connacht regions and is actively spreading from its proposed point of entry near the River Shannon in Foynes, Co. Limerick [73,74]. Building on the recent rodent studies in Ireland [22,79,81,82,83,91] this constant rate of expansion and the presence of uninvaded sites presents the opportunity for long term studies that investigate the establishment and change in parasite communities during a biological invasion and provides natural perturbation experiments for exploring the effects of host diversity.

When studying changes in parasite dynamics during a biological invasion, baseline data are usually absent [20,22]. In the case of Ireland, data collected previously on *M. glareolus*, by Stuart et al. [22] and Loxton et al. [82,83]., can be used as a baseline for future studies investigating changes in parasite communities. Work by Stuart et al. [22] included uninvaded sites as control groups, which will be colonised given the constant expansion rate of *M. glareolus*, demonstrated by White et al. [76], giving clear insight to the changes in parasite communities during the early stages of invasion. Similarly, data collected on core sites can show how parasite dynamics fluctuate with time and previously collected data in expansion front sites showing changes as the population of *M. glareolus* becomes fully established (Table 2). 

It has also been shown that the analysis of helminth dynamics (infection parameters such as prevalence and incidence) is sensitive to the host population structure in collected samples, as parasite species and infection parameters can be dependent on age and functional group [93]. These vary seasonally and in density and phase-dependent ways. To allow us to fully understand the helminth dynamics of this system, this would need to be incorporated into future invasive model studies [22]. 

Further work on immune responses is needed [23], previous studies on rodents have demonstrated that the presence of certain helminth species can influence the presence and intensity of microparasite infections through trade-offs in the Th1 and Th2 immune pathways [66,69]. A review by White and Perkins [90], identified the need for empirical studies analysing immune gene expression during a biological invasion. These authors proposed that the enemy release observed in invasive populations could result in a relaxation of parasite-mediated selection, resulting in changes to the immune phenotype [90]. As mentioned previously, the Irish *M. glareolus* population demonstrates enemy release compared to its native range, but also between the core and invasion front [22,77]. Consequently, the Irish system also presents the opportunity to further investigate trade-offs in *M. glareolus* immune expression and potential associated changes in microparasite communities as their parasite communities change during establishment. 

*A. sylvaticus* population numbers have also been shown to be negatively impacted by the presence of *M. glareolus* in both perturbation studies and in natural settings [87,94]. Figure 2b presents a multi-annual rodent population cycle, similar to the cycling present in the vole population in northern latitudes [44], while the presence of multi-annual cycles has not been thoroughly studied in Ireland, Figure 2c shows the changing population dynamics during a biological invasion, with an increase in one host species, while the other decreases, as *A. sylvaticus* has been shown to be negatively affected by the presence of *M. glareolus* [87]. We propose that just as cycling vole populations have proved a valuable model in the understanding of wildlife parasitology due to their changing host populations, the Irish invasion model also presents unique opportunities to answer novel questions through population changes resulting from the introduction of non-native species. For instance, the recent invasion of *C. russula* has added a potentially novel element to the model system, that is, when a second invader enters the system and causes invasional meltdown [87]. This provides an opportunity to sample *C. russula* in a similar way to how *M. glareolus* has been investigated in the Irish context. Invasive shrews can be sampled from sites where *C. russula* has been introduced the longest, recently invaded and absent. Furthermore, sampling sites where *C. russula* is present with *M. glareolus* and sites where *M. glareolus* is absent could be investigated. 

The recent increase in EIDs has emphasised the need for a pre-emptive approach requiring empirical data on pathogen dynamics [35]. As demonstrated in this review, wild rodent models, globally, have added to our knowledge of parasite communities and ecoimmunology. However, significant gaps remain and the Irish invasion of *M. glareolus* presents a unique opportunity to study these relationships during a biological invasion. Furthermore, the availability of baseline data and the presence of an invasion gradient-consisting of core established sites, recently invaded and uninvaded sites provide a unique opportunity to perform longitudinal and spatiotemporal studies. The Irish model system has the potential to complement current knowledge and allow for further generalities to be identified, which can be adapted to other invasion systems.

## Figures and Tables

**Table 1:** Helminth species recorded in *A. sylvaticus* and *M. glareolus* from selected studies published between 1982–2020. Names in brackets are previous species names.

*A. sylvaticus*				
Taxon	O’Sullivan, Smal and Fairley (1982)	Montgomery and Montgomery (1988; 1990)	Loxton et al. (2016; 2017)	Stuart et al. (2020)
**Nematoda**	*Syphacia stroma* *Capillaria muris-sylvatici* *Trichuris muris* *Heligmosomoides polygyrus* *(Nematospiroides dubius)*	*Syphacia stroma* *Capillaria murissylvatici* *Trichuris muris* *Heligmosomoides polygyrus* *(Nematospiroides dubius)*	*Syphacia stroma* *Aonchotheca murissylvatici* *Trichuris muris* *Heligmosomoides polygyrus*	*Syphacia stroma* *Aonchotheca murissylvatici* *Trichuris muris* *Heligmosomoides polygyrus*
**Cestoda**	*Hymenolepis straminea* *Skrjabinotaenia lobata* *(Catenotaenia lobata)* *Hydatigera taeniaeformis* *(Taenia taeniaeformis)*	*Hymenolepis hibernia* *Skrjabinotaenia lobata* *(Catenotaenia lobata)* *Hydatigera taeniaeformis* *(Taenia taeniaeformis)*	*Hymenolepis hibernia* *Skrjabinotaenia lobata* *Taenia martis* *Hydatigera taeniaeformis* *(Taenia taeniaeformis)*	*Hymenolepis hibernia* *Hymenolepis sp.* *Skrjabinotaenia lobata* *Taenia martis* *Taenia polyacantha* *Hydatigera taeniaeformis*
**Trematoda**	*Brachylaimus recurvum* *Corrigia vitta* *Plagiorchis muris*	*Brachylaima recurva* *Corrigia vitta*	*Brachylaemus recurvum* *Corrigia vitta*	*Brachylaemus recurvum* *Corrigia vitta*
**Total Sp.**	**10**	**9**	**10**	**12**
***M. glareolus***				
**Taxon**	**O’Sullivan, Smal and Fairley (1982)**		**Loxton et al. (2016; 2017)**	**Stuart et al. (2020)**
**Nematoda**	*Capillaria muris-sylvatici* *Trichuris muris*		*Aonchotheca murissylvatici* *Aspiculuris tianjinensis*	*Aonchotheca murissylvatici* *Aspiculuris tianjinensis*
**Cestoda**			*Taenia martis*	*Taenia martis* *Taenia polyacantha*
**Trematoda**	Corrigia vitta			
**Total Sp.**	**3**		**3**	**4**

**Table 2:** Summary of current knowledge, main research question and how this can be addressed using *M. glareolus* as a model system in Ireland.

**Current Knowledge Base:**
Long history of using rodent models in laboratory and wild situations to study disease dynamics worldwide.
Single introduction point and no current eradication attempts.
Presence of baseline parasitological data on the Irish *M. glareolus* biological invasion;
including long established invaded sites, recently invaded sites and uninvaded sites.
**Main Research Questions:**
How parasite dynamics and immune gene expression change during a biological invasion.
**How This Can Be Addressed:**
Data collected during previous studies can be used as a baseline for future studies.
The addition of multiple invaders can further investigate how parasite dynamics change during a biological invasion.
Application of knowledge gained to similar rodent systems elsewhere.

## Data Availability

Not applicable.
